# Holden’s Anatomy: A Manual of Dissection of the Human Body

**Published:** 1885-02

**Authors:** 


### BOOK REVIEWS.

---

HOLDEN'S ANATOMY. *A Manual of Dissection of the Human Body. By* LUTHER HOLDEN, *late President of the Royal College of Surgeons of England; Consulting Surgeon to St. Bartholomew's and the Foundling Hospital. Fifth Edition. Edited by* JOHN LANGTON, *Surgeon to and Lecturer on Anatomy at St. Bartholomew's Hospital; Member of the Board of Examiners, Royal College of Surgeons, England. Octavo, pp. xix, 886. Philadelphia:* P. BLACKISTON, SON & CO., 1885. *Chicago:* JANSEN, McCLURG & CO.

The revised edition of Holden's Anatomy supplies a long-felt want of practitioners and students. The order of dissection has been materially changed, new illustrations have been added, and recent progress in anatomy, more particularly as regards the nervous system and the organs of special sense, has received due attention. As a clear, distinct, concise guide to dissection, the book has no equal in the English language.

